# Cutaneous Larva Migrans Acquired in Brittany, France

**DOI:** 10.3201/eid1511.090261

**Published:** 2009-11

**Authors:** Nienke Tamminga, Wouter F.W. Bierman, Peter J. de Vries

**Affiliations:** Academic Medical Center, Amsterdam, the Netherlands (N. Tamminga, W.F.W. Bierman, P.J. de Vries); University Medical Center Groningen, Groningen, the Netherlands (N. Tamminga); VU University Medical Center, Amsterdam (W.F.W. Bierman)

**Keywords:** Keywords: Cutaneous larva migrans, hookworm infections, zoonoses, France, letter

**To the Editor:** Hookworm-related cutaneous larva migrans is a parasitic dermatosis caused by the penetration of larvae, mostly of a dog or cat hookworm, into the epidermis of humans (*1*,*2*). This eruption is most commonly found in tropical and subtropical areas but was recently reported from western Europe, including Germany (*3*,*4*), England (*5*,*6*), Scotland (*7*), and southern France (*8*). We report a patient from the Netherlands who acquired hookworm-related cutaneous larva migrans while on a holiday in Brittany, France.

A previously healthy 40-year-old man from the Netherlands traveled to Brittany, France, to visit from September 1 to September 15, 2008. He and his partner slept in tents, sometimes camping rough (not on designated camping sites or on private property), and they stayed in low-budget hotels. They spent a lot of time on several beaches along the Atlantic Ocean on the southern shore of Brittany (≈48°N). The weather during their stay was variable. The patient was frequently bitten by mosquitoes, especially on his feet. He had not traveled to the tropics before and did not own any pets.

After his return to the Netherlands, the area around 2 presumed mosquito bites at the lateral side of his right foot became red, swollen, and itchy. This area evolved into a 1-cm pustule that later turned into a bulla. On November 10, he visited his general practitioner, who made a diagnosis of cellulitis and started the patient on amoxicillin/clavulanic acid 625 mg, 3×/day for 10 days. During antimicrobial drug treatment, skin inflammation improved, but after 2 days the patient noticed that an itching red streak had developed, extending from the lesions on the lateral side of the right foot to the whole width of the sole of the foot. The tip of the streak proceeded along the sole of the foot at the rate of 2 cm/day. On the fifth day, he was referred to our Tropical Diseases outpatient clinic.

Physical examination showed 2 elevated, ulcerative lesions on the lateral side of the right foot, and from each originated an elevated serpiginous lesion (Figure, panels B and C). These were typical tortuous lesions 2 cm in width. One of the lesions ran across the whole sole of the right foot and was 14 cm in length (Figure, panels A and C). The medial end of the lesion was fervently erythematous. Based on clinical signs, we diagnosed the skin lesion as hookworm-related cutaneous larva migrans with secondary impetiginization. The patient was subsequently treated with a single oral dose of 12 mg ivermectin. The itch and the progression of the lesion halted instantly and the lesion disappeared during the following weeks. The larva was not extirpated and thus not further identified.

**Figure:**
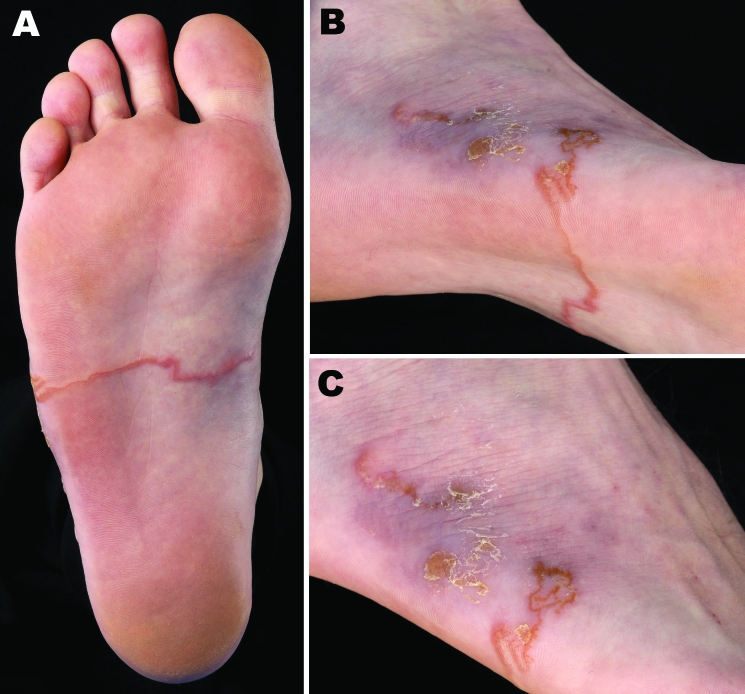
Right foot of a patient from Brittany, France, with a hookworm-related cutaneous larva migrans, showing an elevated serpiginous lesion on the sole of the foot (panels A, B) and ulcerative lesions at the origin of the lesions on the lateral side of the foot (panel C).

Hookworm-related cutaneous larva migrans is usually caused by *Ancylostoma brasiliense*, *A. caninum* or, rarely, *Uncinaria stenocephala*. These zoonotic hookworms need a high temperature and a moist environment to develop from an embryo to filariforme larva (*1*,*2*). Hookworm-related cutaneous larva migrans is typically a disorder of tropical and subtropical zones and it is rather common among tourists who visit tropical beaches. This was the first patient we had seen with this disease who became infected in western Europe. Apart from an exceptionally hot day on August 30 (maximum 26°C), the weather was not particularly warm during the summer of 2008 in Brittany; during the first 2 weeks of September the average minimum and maximum temperatures were 11°C and 17°C, respectively. Rainfall was moderate and humidity was ≈86% (*9*). However, the overall warmer climate, including warmer winters, might have created the conditions for zoonotic hookworm infections in humans in western Europe (*10*).

Our patient may have been infected by *U. stenocephala*, a nematode of dogs that is common in temperate zones but rarely causes hookworm-related cutaneous larva migrans. An increase in ambient temperature might increase the incidence of these zoonotic infections in northern regions. Only 4 cases of hookworm-related cutaneous larva migrans were previously reported in France, all from southern regions (*8*). A northern spread of hookworm-related cutaneous larva migrans could thus point to expansion of the global distribution of the more tropical hookworms or altered conditions that favor the emergence of infection by a zoonotic hookworm such as *U. stenocephala.* Either explanation calls for screening of infection in cats and dogs and preventing pet animals and possibly stray animals from accessing beaches. Clinicians should be aware of the possibility of hookworm-related cutaneous larva migrans in patients who have traveled to western Europe and, in particular, those who have stayed on the beaches.

## References

[1] Heukelbach J, Feldmeier H. Epidemiological and clinical characteristics of hookworm-related cutaneous larva migrans. Lancet Infect Dis. 2008;8:302–9. 10.1016/S1473-3099(08)70098-7 18471775

[2] Hochedez P, Caumes E. Hookworm-related cutaneous larva migrans. J Travel Med. 2007;14:326–33. 10.1111/j.1708-8305.2007.00148.x 17883464

[3] Kienast A, Bialek R, Hoeger PH. Cutaneous larva migrans in northern Germany. Eur J Pediatr. 2007;166:1183–5. 10.1007/s00431-006-0364-0 17216216

[4] Klose C, Mravak S, Geb M, Bienzle U, Meyer CG. Autochthonous cutaneous larva migrans in Germany. Trop Med Int Health. 1996;1:503–4. 10.1046/j.1365-3156.1996.d01-86.x 8765458

[5] Diba VC, Whitty CJ, Green T. Cutaneous larva migrans acquired in Britain. Clin Exp Dermatol. 2004;29:555–6. 10.1111/j.1365-2230.2004.01592.x 15347353

[6] Roest MA, Ratnavel R. Cutaneous larva migrans contracted in England: a reminder. Clin Exp Dermatol. 2001;26:389–90. 10.1046/j.1365-2230.2001.00841.x 11488822

[7] Beattie PE, Fleming CJ. Cutaneous larva migrans in the west coast of Scotland. Clin Exp Dermatol. 2002;27:248–9. 10.1046/j.1365-2230.2002.09852.x 12072020

[8] Zimmermann R, Combemale P, Piens MA, Dupin M, Le Coz C. Cutaneous larva migrans, autochthonous in France. Apropos of a case [in French]. Ann Dermatol Venereol. 1995;122:711–4. 8687062

[9] Weather in Brest. France, from September 1st to 15th, 2008 [cited 2009 Jan 25]. Available from http://weeronline.nl/eurostdf.htm

[10] McMichael AJ, Woodruff RE, Hales S. Climate change and human health: present and future risks. Lancet. 2006;367:859–69. 10.1016/S0140-6736(06)68079-3 16530580

